# Collaborative care in eating disorders treatment: exploring the role of clinician distress, self-compassion, and compassion for others

**DOI:** 10.1186/s40337-023-00741-y

**Published:** 2023-04-06

**Authors:** Josie Geller, Avarna Fernandes, Allison C. Kelly, Lindsay Samson, Suja Srikameswaran

**Affiliations:** 1 St. Paul’s Hospital Eating Disorders Program, 1081 Burrard Street, Vancouver, BC V6Z 1Y6 Canada; 2 Department of Psychiatry, University of British Columbia, Vancouver, Canada; 3 Department of Psychology, University of Waterloo, Waterloo, ON Canada; 4 Department of Psychology, York University, Toronto, Canada

**Keywords:** Collaborative care, Compassion, Self-compassion, Eating disorders, Clinicians

## Abstract

**Background:**

Collaborative care is described as showing curiosity and concern for patient experiences, providing choices, and supporting patient autonomy. In contrast, in directive care, the clinician has authority and the patient is expected to adhere to a treatment plan over which they have limited influence. In the treatment of eating disorders, collaborative care has been shown to be more acceptable and produce better outcomes than directive care. Despite widespread patient and clinician preference for collaborative care, it is common for clinicians to be directive in practice, resulting in negative patient attitudes toward treatment and poor adherence. There is a need to understand factors which contribute to its use.

**Purpose:**

This study examined the contribution of clinicians' experience of distress and how they relate to themselves and others in times of difficulty (self-compassion and compassion for others), to their use of collaborative support.

**Method:**

Clinicians working with individuals with eating disorders from diverse professional backgrounds (*N* = 123) completed an online survey.

**Results:**

Whereas clinician distress was not associated with use of collaborative or directive support behaviours, self-compassion and compassion for others were. Regression analyses indicated that compassion for others was the most important determinant of collaborative care.

**Discussion:**

Relating to their own and others’ distress with compassion was most important in determining clinicians’ use of collaborative support. Understanding how to cultivate conditions that foster compassion in clinical environments could promote the delivery of collaborative care.

## Abstract (plain english summary)

Collaborative care involves showing curiosity and concern for patients’ wellbeing regardless of their adherence to treatment, providing choices, and supporting the patient in deciding what is best for them. Although collaborative care has been shown to contribute to better treatment outcomes than directive care, it is common for clinicians to be directive, resulting in negative attitudes toward treatment and lower levels of adherence. A factor that may contribute to the care clinicians provide is their experience of distress and how they respond to their own and others’ distress in times of difficulty. In this study, self-compassion and compassion for others were associated with greater use of collaborative care, with compassion for others being the stronger predictor. Clinicians’ own experience of distress was not related to the manner in which they offered support. Fostering compassion in clinical environments could support the delivery of collaborative care.

## Introduction

Collaborative care is emphasized in evidence-based clinical practice, and involves a focus on patient autonomy, the provision of choices, and showing care and concern for patients’ well-being, independent of patients’ adherence to treatment [1, 2]. In Cognitive Behaviour Therapy (CBT; [3]) and Dialectical Behaviour Therapy (DBT; [4]), collaboration is underscored in its encouragement of teamwork and active patient participation. A meta-analysis of the relations between collaboration and outcome in CBT for anxiety disorders suggests that even within a treatment model emphasizing collaboration, the degree to which this stance is applied is related to greater symptom improvement [5]. The benefits of a collaborative approach may be attributed to patients’ enhanced well-being and engagement through their involvement in treatment decision-making and in the ability to capitalize upon existing competencies [6, 7].

The benefits of a collaborative approach are especially pronounced in individuals with eating disorders (EDs), where low motivation is common [8, 9] and is a key predictor of treatment outcome [10]. Collaborative treatment approaches in this population have been linked to higher motivation for change [11, 12], greater treatment satisfaction and adherence [13, 14] and better patient outcomes [15, 16]. However, despite evidence on the effectiveness of this approach and the beliefs of clinicians, patients and carers that a collaborative stance is most helpful [16, 17], it is common for clinicians and carers to be directive in practice. A directive approach is operationalized as using demanding statements that inhibit patient autonomy, and unsolicited opinions that may be experienced as judgmental or critical [17]. In two studies using coded responses to written vignettes depicting difficult situations with an individual with an ED, the majority of carer responses (60% and 76%, respectively) were coded as directive [17, 18]. Thus, despite a universal preference for collaborative care, carers and clinicians are prone to using a directive approach. Understanding factors that enhance the use of a collaborative stance in practice is needed in order to facilitate patient readiness, engagement and satisfaction with the care they receive, and increase the likelihood of patients benefitting from treatment.

The investigation of process variables (i.e., the way treatment is delivered rather than the content of the treatment) is an emerging area of inquiry, with research focusing on collaboration in carers, or loved ones of individuals with EDs. Use of a collaborative stance in carers has been linked to relationship factors (e.g., perceiving the ED as having a less negative effect on the family), interpersonal style (e.g., being less vindictive and cold) and beliefs about the benefits of collaboration [18]. Unlike carers, clinicians do not have a history with patients that affect their interactions, and are more likely to be perceived as authorities in the recovery process. Clinicians are also involved in treatment decisions, such as deciding on the non-negotiable aspects of care (e.g., weight gain expectations), which may be associated with strong negative feelings in patients that in turn affect clinicians’ level of distress and emotional well-being. Finally, clinicians may experience distress because they feel responsible for the patient’s recovery and physical health.

Possibly, clinicians’ experience with their own and their patients’ distress impacts the extent to which they are collaborative. To explore this hypothesis, three aspects of clinicians’ experience were examined: their personal distress levels, the way they relate to their own distress (self-compassion), and the way they relate to the distress of others (compassion for others).

Regarding clinicians’ own distress, the effects of stress on performance in health care are well documented, with elevated clinician stress levels impeding performance on tasks that require complex thought and attention [19, 20]. Not surprisingly, working in fields that involve a high level of psychological demand, such as mental health, is associated with high rates of burnout [21, 22]. Distress may interfere with clinicians’ ability to practise a collaborative stance by reducing their capacity to be curious and open, and stay committed to a collaborative care philosophy.

The way clinicians relate to distress may also impact their caregiving style. Compassion has been described as a sensitivity to distress, with the desire to respond in a manner that alleviates suffering [23, 24]. Self-compassion in health care providers has been associated with positive personal qualities, including greater well-being, mindfulness, and resilience, as well as lower levels of burnout and compassion fatigue (e.g., [25–27]). Mindfulness training for health care providers is linked to improvements in self-compassion, and is also associated with enhanced confidence in providing compassionate care [28]. Self-compassion may assist in the delivery of collaborative care by allowing clinicians to attend to their distress and practise self-care, thus supporting their ability to maintain a collaborative stance.

Finally, compassion for others has also been associated with positive relationship factors in medical practice [29] and may impact the delivery of collaborative care. There is some evidence to support this hypothesis including clinicians rating the ability to demonstrate emotional resonance as the most important quality of exemplary care provision [30, 31]. Additionally, physicians who report higher levels of compassion provide more meticulous care that is in turn associated with more positive patient experiences [32]. Clinicians’ compassion for their patients may foster greater empathy, leading them to offer care that is aligned with how they themselves wish to be treated [18].

Given the benefits of collaborative care, increasing understanding of factors that promote use of this stance in health care settings is needed. In this study, it was hypothesized that lower levels of clinician distress and higher levels of self-compassion and compassion for others would be associated with the delivery of collaborative care.

## Methods

### Participants

Participants were recruited from national and provincial membership lists of ED clinicians, and 123 Canadian clinicians who identified themselves as having experience working with individuals with EDs participated in an online survey. One hundred and ten participants (90%) identified as female, six (5%) identified as male and seven (6%) did not specify their gender. The mean sample age was 41.99 years (*SD* = 10.87). Ninety-nine participants (80%) identified as Caucasian, seven (6%) as Asian, three (2%) as First Nations or Metis, and, two as Latino, African and Middle Eastern, respectively (5%). Eight participants (7%) did not specify their ethnic background. Healthcare settings were community (*n* = 56; e.g., private practice) and intensive treatment (*n* = 44; e.g. inpatient program). Ten participants described their setting as “other.” The sample consisted of psychologists/counsellors (*n* = 35), dieticians (*n* = 34), social workers (*n* = 13), nurses (*n* = 10), occupational therapists (*n* = 5), physicians (*n* = 7), and other health care workers (*n* = 6).

### Measures

#### Depression anxiety and stress scales-short form (DASS; [33])

The 21-item DASS (Short Form) consists of three scales assessing depression, anxiety and stress levels over the past week; the stress scale was used for the current study. The DASS demonstrates adequate construct validity and good internal consistency [34, 35]. Internal consistency in this study was α = 0.82 for stress.

#### Compassionate engagement and action scales for self and others (CEAS; [36])

The CEAS consists of three 10-item scales measuring self-compassion, compassion for others, and compassion from others. Each scale is comprised of items measuring the ability to be sensitive to suffering, and the motivation to respond to, and alleviate distress; the Compassionate Self and Compassionate Others scales were utilized. The CEAS has good psychometric properties [36] and in this study, internal consistencies were α = 0.89 for self-compassion, and α = 0.84 for compassion for others.

#### Health care climate questionnaire—short form (HCCQ; [37])

This 7-item measure assesses perceived autonomy support, or collaborative care, in healthcare settings. It was modified by Zuroff et al. [38] with the addition of two items from the original HCCQ [39] for a more comprehensive assessment in mental health treatment settings. In this research, further modifications were made for use with a clinician sample (originally developed for patient samples). The HCCQ demonstrates excellent structural and construct validity [40] and its internal consistency in this sample was α = 0.78.

#### Support behaviours scale (SBH; [1])

This 19-item measure assesses support behaviors in carers of individuals with EDs. Modifications were made for use with a clinician sample. The SBH includes two collaborative support behaviour subscales, Concerned (e.g., “recognizing the difficulty of their situation”) and Encouraging (e.g., “ask if they want help with their symptoms”), and one Directive behaviour subscale (e.g., “criticizing eating behaviour”). In this study, internal consistencies for the Concerned, Encouraging, and Directive subscales were α = 0.85, α = 0.88, and α = 0.82, respectively.

### Analysis plan

Zero-order correlations were conducted among all study variables to determine relations among support behaviours, self-compassion, compassion for others, and distress. To ensure the detection of meaningful associations while balancing concerns about Type 1 error, alpha was set at *p* < 0.01 in interpreting statistical significance. To determine clinician factors most important in their use of collaborative support, hierarchical multiple regression analyses (forward entry) were conducted, for each of the four collaborative support behaviours, while controlling for clinician age.

## Results

### Correlation analyses

Zero-order correlations examined relations among study variables. Compassion for others and self-compassion were significantly associated with collaborative support behaviours (HCCQ, SBH Encouraging, and SBH Concerned) and were not significantly associated with directive support behaviours (Table 1). DASS stress scores were not significantly associated with collaborative or directive support behaviours.

**Table 1:** Zero-order correlations among study variables

	HCCQ autonomy	SBH encouraging	SBH concerned	SBH directive	Compassion for self	Compassion for others	DASS—stress
HCCQ autonomy	–	0.45***	0.35***	− 0.15	0.35***	0.44***	− 0.12
SBH encouraging		–	0.49***	0.19	0.29**	0.43***	− 0.18
SBH concerned			–	0.09	0.29**	0.48***	− 0.07
SBH directive				–	− 0.06	0.04	− 0.02
Compassion for self					–	0.56***	− 0.24*
Compassion for others						–	− 0.13
DASS—stress							–

HCCQ, Healthcare Climate Questionnaire; SBH, support behaviours; DASS, Depression Anxiety and Stress Scale

*p < .05 (not interpreted); **p < .01; ***p < .001

### Multiple regression analyses

Hierarchical multiple regression analyses were conducted for each of the collaborative and directive support measures (HCCQ, SBH Encouraging, SBH Concerned, SBH Directive) using the three predictor variables (DASS stress, CEAS compassion for self, and CEAS compassion for others), controlling for clinician age. After controlling for age, only higher compassion for others contributed to all three collaborative support behaviour regressions (Table 2). A regression for directive support behaviours was not conducted as none of the correlations with the predictor variables were significant.

**Table 2:** Multiple regression models predicting collaborative care from distress, self-compassion, and compassion for others

	Autonomy support			Collaborative—encouraging			Collaborative—concerned		
	*R*^2^	*F*	*B*	*R*^2^	*F*	*B*	*R*^2^	*F*	*B*
Step 1	.06	4.82*		.04	2.87		.02	1.44	
Age			.21			.19			.14
Step 2	.20	8.01***		.19	8.53***		.23	11.15***	
Distress			–			–			–
Self-compassion			–			–			–
Compassion for others			.35**			.39***			.47***

*p < .05, **p < .01, ***p < .001

## Discussion

In this research, three aspects of ED clinicians’ experience of distress and their delivery of collaborative care were examined. Clinician distress was not associated with use of collaborative or directive support. However, relating to their own and others’ distress with compassion was associated with greater use of a collaborative support stance. Interestingly, when the relative contribution of self-compassion and compassion for others was examined in multiple regression analyses, only compassion for others was significantly associated with collaborative support behaviours.

The finding of a significant association between compassion for self and others and use of a collaborative stance could be explained by the role that the practice of compassion plays in distress tolerance and affect regulation [41, 42]. Possibly, compassion allows clinicians to stay present when difficult emotions arise in themselves and their patients. Self-compassion may be helpful by enabling them to tolerate their own distress in difficult encounters, remaining focused on what serves the situation, and refraining from imposing a solution. Indeed, there is evidence that self-compassion represents key mechanisms in emotion regulation in mood and anxiety disorders [43]. Conversely, compassion for others may help clinicians to understand and join with their patients in times of difficulty, validate their patients’ needs, and consider options that are tailored to each patient. This research suggests that compassion for others plays a more important role in determining the provision of collaborative support, perhaps because one’s sensitivity to the suffering of others enhances curiosity about how best one can help, and is most relevant to maintaining a non-directive, collaborative stance.

The lack of association between clinicians’ reports of distress and their use of a collaborative approach suggests that clinicians are capable of supporting patients in a collaborative manner independent of their own personal difficulties. Possibly, experience and training, and the temperament of individuals who choose to work in healthcare and more specifically in the field of eating disorders, contribute to their ability to remain focused on patients and set their own needs aside when experiencing personal difficulties. It is also possible that situational distress resulting from stressors in the workplace, as opposed to general distress over the previous week (as assessed in this study), has a more significant impact on clinicians’ collaboration.

Interestingly, neither clinician distress nor compassion for self or others were related to directive support. Previous research in carers has shown that believing that a directive support stance is helpful was associated with directive behaviours [18]. Further research is required to determine whether similarly, clinicians’ beliefs about directive support are associated with directive behaviours.

The correlational design of the study limits causality inferences from being made, and the sample size was not large enough to examine differences across subgroup populations. Findings were also limited by the self-report design and use of questionnaires to assess collaborative behaviours. Vignettes or in-vivo observations would have allowed for a more realistic portrayal of clinician behaviours.

## Conclusions

This research supports training in compassion for health care providers. Compassion-focused educational interventions have been piloted for trainees and health care professionals and have been associated with improvements in clinicians’ knowledge, skill and confidence in practicing compassion in their work (e.g., [44–46]). For instance, the Self-Compassion for Healthcare Communities (SCHC) program is an adaptation of the empirically-supported Mindfulness Self-Compassion program created by Neff and Germer [47], modified for the healthcare context [48]. This program consists of teaching self-compassion, and providing opportunities to practise meditation, and brief exercises to cultivate compassion for self and others on the job. Participating in SCHC has been associated with improvements in psychological outcomes, including improved mindfulness, resilience, and reductions in stress, burnout and depression [48, 49]. These promising results suggest that compassion interventions are acceptable and effective in the health care context, and future research is needed to determine whether such interventions also positively impact clinical practice and the delivery of collaborative care.

## Data Availability

The data that support the findings of this study are available from the corresponding author upon reasonable request.

## Abbreviations

CBT: Cognitive behaviour therapy
ED: Eating disorder
DASS: Depression Anxiety and Stress Scale
CEAS: Compassionate Engagement and Action Scales for Self and Others
HCCQ: Healthcare Climate Questionnaire
SBH: Support Behaviours Scale
SCHC: Self-Compassion for Healthcare Communities
